# Schisandrin Protects against Norepinephrine-Induced Myocardial Hypertrophic Injury by Inhibiting the JAK2/STAT3 Signaling Pathway

**DOI:** 10.1155/2021/8129512

**Published:** 2021-06-16

**Authors:** Min Yang, Xing-Can Jiang, Lei Wang, Dong-An Cui, Jing-Yan Zhang, Xu-Rong Wang, Hai-Peng Feng, Kang Zhang, Kai Zhang, Jian-Xi Li, Xue-Zhi Wang

**Affiliations:** ^1^Engineering & Technology Research Center of Traditional Chinese Veterinary Medicine, Gansu, Lanzhou, China; ^2^Lanzhou Institute of Husbandry and Pharmaceutical Sciences, Chinese Academy of Agricultural Sciences (CAAS), Lanzhou, China

## Abstract

*Aims*. Heart failure is closely associated with norepinephrine-(NE-) induced cardiomyocyte hypertrophy. Schisandrin is derived from the traditional Chinese medicine Schisandra; it has a variety of pharmacological activities, and the mechanism of schisandrin-mediated protection of the cardiovascular system is not clear. *Main Methods*. NE was used to establish a cardiomyocyte hypertrophy model to explore the mechanism of action of schisandrin. An MTT assay was used for cell viability; Hoechst fluorescence staining was used to observe the cell morphology and calculate the apoptosis rate. The cell surface area was measured and the protein to DNA ratio was calculated, changes in mitochondrial membrane potential were detected, and the degree of hypertrophic cell damage was evaluated. WB, QRT-PCR, and immunofluorescence were used to qualitatively, quantitatively, and quantitatively detect apoptotic proteins in the JAK2/STAT3 signaling pathway. *Key Findings*. In the NE-induced model, schisandrin treatment reduced the apoptosis rate of cardiomyocytes, increased the ratio of the cell surface area to cardiomyocyte protein/DNA, and also, increased the membrane potential of the mitochondria. The expression of both JAK2 and STAT3 was downregulated, and the BAX/Bcl-2 ratio was significantly reduced. In conclusion, schisandrin may protect against NE-induced cardiomyocyte hypertrophy by inhibiting the JAK2/STAT3 signaling pathway and reducing cardiomyocyte apoptosis.

## 1. Introduction

Schisandrin is one of the dibenzocyclooctadiene lignans in Schisandra and has antioxidant, anti-inflammatory, antiapoptotic effects [1–5]. Studies have found that Schisandra and lignans have potentially beneficial effects on the treatment of cardiovascular diseases. Currently, research reports have found that *Schisandra chinensis* extracts schisandrin B, gomisin N, schisandrin A, and schisandrin C exert various pharmacological activities for the treatment of cardiovascular disease and have studied the molecular mechanism of action [6]. The structure of schisandrin is similar to that of schisandrin A. Schisandrin may play a role in the treatment of cardiovascular disease.

Heart failure is associated with chronic increases in plasma NE and mast cell numbers, and increased NE is a key marker of pathological heart failure [7–11]. Thus, blocking NE is conducive to cell survival and also inhibits cardiac hypertrophy [10]. Modern studies have shown that JAK2/STAT3 signaling pathway activation is associated with cardiac hypertrophy [12–14]; it is one of the important pathways of cardiac apoptosis [15] and is of great significance in cardiomyocyte signal transduction pathways. JAK2 is considered to be a new treatment target for inhibiting the progression of hypertrophy to heart failure. Studies have found that STAT3 activation occurs in cardiac hypertrophy and is involved in cell growth. Key regulators may be important regulators of cardiovascular system growth [13]. In this study, based on the pharmacological activity of schisandrin and the important significance of JAK2/STAT3 in the regulation of cardiomyocyte hypertrophy, we pretreated H9C2 cardiomyocytes with schisandrin and induced cardiomyocyte hypertrophy with NE. We found that the JAK2/STAT3 signaling pathway was inhibited, the BAX/Bcl-2 ratio was decreased, the mitochondrial membrane potential was stabilized, the cardiomyocytes were protected, and apoptosis was inhibited.

## 2. Materials and Methods

### 2.1. Passage and Culture of Rat H9C2 Cardiomyocytes

H9C2 cells were from the Cell Bank of the Chinese Academy of Sciences [16], maintained in Dulbecco's modified Eagle's medium (DMEM; USA) containing 10% fetal bovine serum (Gibico; USA) at 37°C with 5% CO2. The cells were rinsed in phosphate-buffered saline (PBS) and treated with 0.25% trypsinat 37°C (Gibico, USA) for passaging cells.

### 2.2. Methyl Thiazolyl Tetrazolium (MTT) Assay

Cells with a density of 1 × 10^5^ cells/mL per well were inoculated into 96-well plates. After 24 h, in the positive drug group [17], a DMEM complete culture medium containing 5 *μ*g/mL was added to each well with captopril [15, 18], and a schisandrin-DMEM complete cell culture medium containing 5 *μ*g/mL, 10 *μ*g/mL, and 20 *μ*g/mL was added to each well in the low-, middle-, and high-dose groups, respectively. A certain concentration of norepinephrine can induce cardiac hypertrophy [19, 20]. After 48 hours culturing, the complete culture medium of DMEM containing 10^−6^ mol/L NE was added to each well of the model group, positive drug group, low-, middle-, and high-dose groups of schisandrin for 48 hours, and pictures were taken under the microscope (Olympus, Tokyo, Japan) to observe the morphological changes of the cells (scale bar: 500 *μ*m). Cell viability was evaluated by the 3-(4, 5-dimethylthiazol-2-yl)-2, 5-diphenyl tetrazolium bromide (MTT) assay based on the reduction of soluble yellow tetrazolium compound into insoluble purple formazan crystals produced in living cells. After the treatment procedure, MTT was added to each well and incubated for 4 h at 37°C in darkness. The supernatant was removed, and then, 150uL dimethyl sulfoxide was added to dissolve the formazan compound. The absorbance was measured at 490 nm using a microplate reader. The H9C2 survival rate was calculated by the following formula:

(1) $$\begin{array}{c} \text{H}9\text{C}2\text{ survival rate}\left(\%\right)=\frac{\text{OD value of treated group}}{\text{OD value of control group}}\times 100\%. \end{array}$$

Measurement of the cell surface area: the cardiomyocytes were cultured in a 24-well plate, and 6 wells of the three groups of cells were cultured in parallel. Photographs were taken using a phase contrast microscope (Leica DMi1, Beijing, China) with a 20× objective (scale bar: 25 *μ*m). ImageJ software was used to process the images, and the surface area of more than 100 cardiomyocytes in each field of view was measured and counted.

Measurement of the protein/DNA ratio: the adherent cells were digested with 0.25% trypsin to prepare a cell suspension, and the cells were processed using an automated cell counter (Nexcelom Bioscience) for cell counting. Then, the cells were lysed with M-PER cell lysis buffer (Thermo Fisher Scientific, USA); the lysate was centrifuged, and the supernatant was collected. Total protein detection was performed according to the instructions of the Micro BCA Protein Assay Kit (Thermo Fisher Scientific, USA). The DNA content was determined using a Green II dsDNA Quantitation Kit Plus (Yu heng, Su zhou, China), and the DNA concentration in the sample was further determined based on the generated standard curve. Finally, the ratio of protein/DNA was calculated.

### 2.3. Hoechst 33342 Staining

After the cells were treated with drugs, the cell culture solution was sucked away, and the cells were washed with PBS and fixed in a 4% cell fixation solution (Solarbio, Beijing, China) at room temperature for 20 min. Hoechst 33342 fluorescent dye (5 *μ*g/mL; Biyuntian, Shanghai, China) was added to each well, and the plate was incubated at 37°C for 20 minutes. Subsequently, the cells were washed with PBS and photographed with a universal fluorescence inverted microscope (OLYMPUS IX71, Japan) (scale bar: 25 *μ*m). Fifty visual fields were randomly selected in each group. The number of apoptotic cells and the total number of cells were recorded. The H9C2 apoptotic rate was calculated by the following formula:

(2) $$\begin{array}{c} \text{H}9\text{C}2\text{ apoptotic rate}\left(\%\right)=\frac{\text{Apoptotic number of H}9\text{C}2}{\text{Total number of H}9\text{C}2}\times 100\%. \end{array}$$

### 2.4. Mitochondrial Membrane Potential Staining

The cells were stained with a JC-1 mitochondrial membrane potential detection kit (Biyuntian, Beijing, China) according to the manufacturer's instructions. The CCCP group was used as positive control to induce mitochondrial membrane potential decline. JC-1 staining solution was added to cell culture medium and mixed well. The cells were incubated with the stain at 37°C for 20 min. Then, the supernatant was discarded, and the cells were washed twice with JC-1 staining buffer (1×) and photographed under a laser scanning confocal microscope (ZEISS, Shanghai, China) (scale bar: 25 *μ*m). A fluorescence microplate reader was used to detect the fluorescence intensity; the excitation wavelength was set at 490 nm, and the emission wavelength was set at 530 nm to detect the JC-1 monomer. The excitation wavelength was set at 525 nm, and the emission wavelength was 590 nm to detect the JC-1 polymer.

### 2.5. Immunofluorescence

The cells were fixed in 4% paraformaldehyde (Solarbio, Beijing, China) for 20 min, washed with PBS, and permeabilized in 0.5% Triton X-100 for 15 min. Subsequently, the cells were treated with 5% BSA at room temperature for 1 h. The cells were then incubated with the corresponding dilution ratio of the primary antibody overnight at 4°C. Then, the cells were washed with PBST and incubated with diluted fluorescent secondary goat anti-rabbit IgG H&L (FITC; 1 : 500 diluted; Abcam, USA) and conjugated goat anti-rabbit IgG H&L (1 : 500 diluted; Proteintech, USA) antibodies at 37°C for 1 hour. Subsequently, the cells were stained with 5 *μ*g/mL DAPI solution for 15 min at 37°C. A fluorescence microscope (ZEISS, Shanghai, China) was used to observe the cells and collect pictures (scale bar: 25 *μ*m).

### 2.6. Western Blot (WB) Assay

The cells in each group were lysed in M-PER cell lysis buffer (Thermo Fisher Scientific, USA). The total protein of each group was collected and quantified measuring with a BCA total protein kit (Thermo Fisher Scientific, USA). Proteins of the same volume were separated by SDS-PAGE. Then, the proteins were transferred to the semidry membrane. Subsequently, membranes were blocked in 5% BSA for 1 hour at room temperature and incubated with *β*-actin (1 : 2500, Proteintech), rabbit anti-BAX (1 : 250, Proteintech), rabbit anti-Bcl-2 (1 : 1000, Abcam), rabbit anti-JAK2 (1 : 1000, Cell Signaling Technology), and rabbit anti-STAT3 (1 : 1000, Cell Signaling Technology) antibodies at 4°C overnight. Subsequently, the membranes were incubated with secondary antibodies labeled with horseradish peroxidase (HRP) for 2 hour. The membrane was exposed to the developing solution of electrogenerated chemiluminescence (ECL) (Advansta, CA, USA). The optical density of protein bands was analyzed by ImageJ image analysis software.

### 2.7. Quantitative Real-Time PCR

Quantitative real-time PCR (QRT-PCR) was performed for the quantitative detection of mRNA. Total RNA was extracted using a High-purity Total RNA Rapid Extraction Kit (BIOTEKE CORPORATION, Beijing, China). RNA is transcribed into cDNA with a PrimeScriptTMRT reagent Kit with a gDNA Eraser (Takara, Dalian, China). QT-PCR was performed using a QuantStudio 5 real-time PCR instrument (Thermo Fisher Scientific, USA) and TB GreenTM Premix Ex TaqTM II (Takara, Dalian, China). The primer sequences for qRT-PCR were as follows: *β*-actin mRNA, 5′-GGAGATTACTGCCCTGGCTCCTAGC-3′ (forward), 5′-GGCCGGACTCATCGTACTCCTGCTT-3′ (reverse); JAK2 mRNA, 5′-TCATAAACCTGGAGACCCT-3′ (forward), 5′-ATGTTTCCCTCTTGACCAC-3′ (reverse); STAT3 mRNA, 5′-TAACATTCTGGGCACGAACA-3′ (forward), 5′-GGCATCACAATTGGCACGG-3′ (reverse); Bax mRNA, 5′-GGCTGGACACTGGACTTCCT-3′ (forward), 5′-GGTGAGGACTCCAGCCACAA-3′ (reverse); and Bcl-2 mRNA, 5′-ACGGTGGTGGAGGAACTCTT-3' (forward), 5′-GCAGATGCCGGTTCAGGTA-3′ (reverse). The Ct values obtained for each sample were calculated. The results were analyzed according to the 2-ΔΔCt method. *β*-Actin was used as an internal reference gene.

### 2.8. Statistics

The experimental data were analyzed using SPSS 19.0 (SPSS, Inc., USA). The data are expressed as the mean ± standard deviation (SD). A *t*-test was used for comparisons between groups. The different symbols in the table represent *P* < 0.05, and the difference was considered statistically significant.

## 3. Results and Discussion

### 3.1. Schisandrin Increases the Viability of Cardiomyocytes

**Figure 1:**
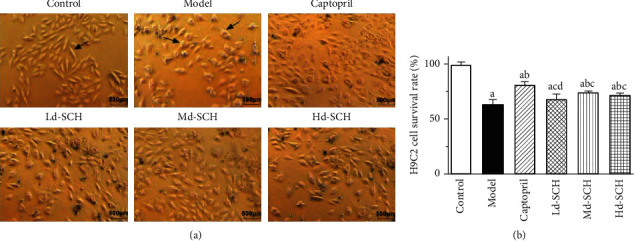
(a) (Scale bar: 500 *μ*m) morphological changes of myocardial cells in each group were studied; control: H9C2 cells growth without any treatment; model: norepinephrine-induced myocardial hypertrophic injury and without any treatment. Coptopril: H9C2 cells were pretreated with captopril. Different dosages of SCH were administrated in the cell model with myocardial hypertrophic injury (Ld-SCH, Md-SCH, and Hd-SCH). (b) The applied MTT assay for the determination of the effect of schisandrin pretreatment on NE-treated cardiomyocyte survival. SCH, schisandrin; Ld, low dose; Md, medium dose; Hd, high dose; a, *P* < 0.05 vs. the control group; b, *P* < 0.05 vs. the model group; c, *P* < 0.05 vs. the captopril group; and d, *P* < 0.05 vs. the medium-dose schisandrin group.

Figure 1 shows the changes in cardiomyocyte morphology and cell viability in different groups, the H9C2 cardiomyocytes of the control group showed a long spindle shape, and the cardiomyocytes of the model group increased in volume, mostly triangular and elliptical (Figure 1(a)). Compared with the control group, the survival rate of cardiomyocytes in the model group was significantly reduced (*P* < 0.05). Compared with that of the model group, the cell viability of the captopril group and schisandrin medium- and high-dose groups was significantly increased (*P* < 0.05), but there was no significant difference in the low-dose schisandrin group (*P* > 0.05), indicating that captopril and schisandrin can protect cardiomyocytes and increase the viability of H9C2 cells (Figure 1(b)).

### 3.2. Hoechst Staining Assay

**Figure 2:**
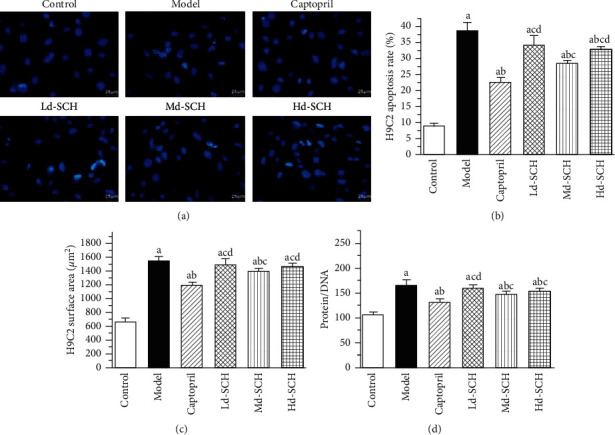
Detection of the apoptosis rate by Hoechst fluorescence staining (scale bar: 25 *μ*m). (a) Morphological changes of nuclei assessed by Hoechst fluorescence staining. (b) Statistical chart of the apoptosis rate of myocardial cells in each group. (c) Statistical graph of changes in the surface area of myocardial cells in each group. (d) Statistical graph of changes in the protein/DNA ratio of cardiomyocytes in each group. Ld, low dose; Md, medium dose; Hd, high dose; a, *P* < 0.05 vs. the control group; b, *P* < 0.05 vs. the model group; c, *P* < 0.05 vs. the captopril group; and d, *P* < 0.05 vs. the medium-dose schisandrin group.

Hoechst fluorescence staining was used to detect the apoptotic morphology and apoptosis rate of H9C2 cardiomyocytes, as shown in Figure 2. The nuclei of the control group were clearly visible, the morphology was normal, and the blue fluorescence was normal. Bright blue fluorescence appeared in the model group, the nuclear morphology was incomplete, and there were irregular shape changes, such as crescent shapes. Compared with that of the control group, the apoptosis rate of the other six groups was decreased (*P* < 0.05). Compared with that of the model group, the apoptosis rate of the medium- and high-dose schisandrin groups and the captopril group was significantly reduced (*P* < 0.05), but there was no significant change in the low-dose schisandrin group (*P* > 0.05). Compared with that of the captopril group, the apoptosis rate of the schisandrin medium-dose group did not increase significantly (*P* < 0.05). These results indicate that schisandrin and captopril both protect cardiomyocytes.

### 3.3. Schisandrin Improves Cardiomyocyte Hypertrophy Induced by NE

By measuring changes in the myocardial cell surface area and protein/DNA ratio, the degree of myocardial hypertrophy was further determined (Figure 2); compared with that of the model group, the myocardial cell surface area of the captopril group and the medium-dose schisandrin group was significantly decreased (*P* < 0.05). Compared with that of the captopril positive-control group, that of the medium-dose schisandrin group was not significantly increased (*P* > 0.05). The medium dose of schisandrin and 5 *μ*g/mL captopril had similar effects on the surface area of cardiomyocytes treated with NE. Furthermore, 10 *μ*g/mL schisandrin had the greatest effect on the promotion of myocardial cell surface area recovery. We observed the protein/DNA ratio and found that the ratio of protein/DNA was significantly increased under 10^−6^ mol/L NE induction conditions (*P* < 0.05 compared with the control group). After pretreatment with schisandrin and captopril, the ratio of protein/DNA decreased, but the change in the captopril and schisandrin groups was the most significant (*P* < 0.05). This finding indicates that schisandrin has a protective effect against NE-induced cardiomyocyte hypertrophy.

### 3.4. Mitochondrial Membrane Potential Assay

**Figure 3:**
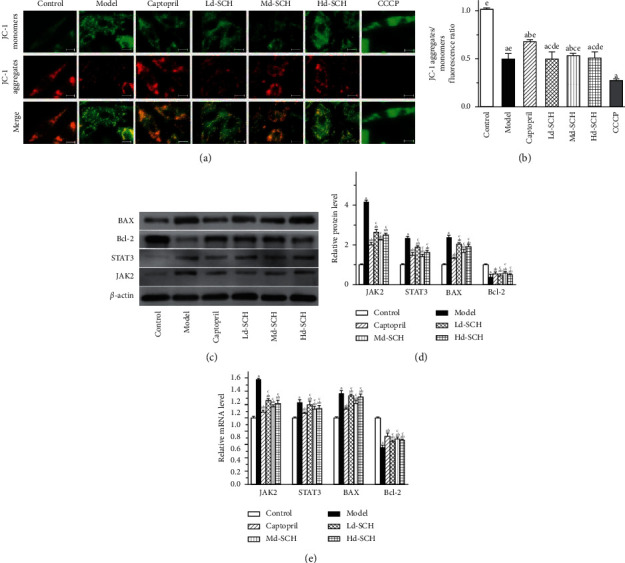
(a) Fluorescence picture of mitochondrial membrane potential changes (scale bar: 25 *μ*m). (b) Red fluorescence intensity/green fluorescence intensity ratio statistics. (c) Band diagram of *β*-actin, JAK2, STAT3, BAX, and Bcl-2 protein expression detected by WB. (d) Statistical comparison of the gray value of JAK2/*β*-actin, STAT3/*β*-actin, BAX/*β*-actin, and Bcl-2/*β*-actin proteins. (e) The statistical value of the ratio of JAK2/*β*-actin, STAT3/*β*-actin, BAX/*β*-actin, and Bcl-2/*β*-actin mRNA. Ld, low dose; Md, medium dose; Hd, high dose; a, *P* < 0.05 vs. the control group; b, *P* < 0.05 vs. the model group; c, *P* < 0.05 vs. the captopril group; d, *P* < 0.05 vs. the medium-dose schisandrin group; and e, *P* < 0.05 vs. the CCCP group.

The changes in mitochondrial membrane potential are shown in Figure 3. The mitochondrial membrane potential of the experimental group was significantly increased compared with that of the CCCP group (*P* < 0.05). In the comparison of the model groups, the membrane potential of the captopril group and the medium-dose schisandrin group decreased (*P* < 0.05). In the low-dose schisandrin group, there was no significant change in mitochondrial membrane potential (*P* > 0.05). Compared with that of the captopril group, there was no obvious difference in the medium-dose schisandrin group (*P* > 0.05), and the mitochondrial membrane potential was similar to that of the captopril group.

### 3.5. Western Blot and Quantitative Real-Time PCR Assays

The results of the WB and RT-PCR are shown in Figure 3. Compared with that of the control group, the relative expression of JAK2 and STAT3 protein and mRNA in the model group was increased (*P* < 0.05). Compared with the model group, the schisandrin and captopril groups all displayed decreased relative protein and mRNA expression of JAK2 and STAT3; the medium-dose schisandrin group displayed the most significant decrease (*P* < 0.05). The expression in the captopril control group was similar to that of the schisandrin group. Compared with that in the control group, the relative expression of BAX and Bcl-2 protein and mRNA in each experimental group was downregulated (*P* < 0.05). Compared with the model group, the medium-dose schisandrin group displayed significantly upregulated BAX protein and mRNA expression (*P* < 0.05) and downregulated Bcl-2 protein and mRNA expression (*P* < 0.05). There was no significant difference in the BAX and Bcl-2 protein and mRNA expression in the captopril group and the medium-dose schisandrin group. We found that the gene expression trend detected by RT-PCR was consistent with the protein expression in the WB, indicating that schisandrin inhibits the JAK2/STAT3 pathway, downregulates BAX expression, and upregulates Bcl-2 expression.

### 3.6. Immunofluorescence Assay

**Figure 4:**
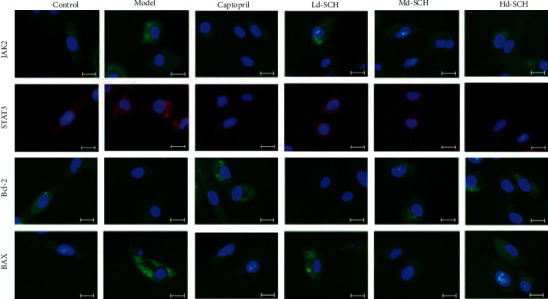
Immunofluorescence assay showing the expression of JAK2, STAT3, Bax, and Bcl-2 in cells of each experimental group (scale bar: 25 *μ*m).

The expression of JAK2, STAT3, BAX, and Bcl-2 was detected by immunofluorescence (Figure 4), and JAK2, STAT3, BAX, and Bcl-2 were positively expressed in the cytoplasm. The fluorescence intensity of JAK2 and STAT3 was the highest in the model group and the lowest in the control group. After pretreatment with low, medium, and high doses of schisandrin, the fluorescence intensity decreased accordingly. In the immunofluorescence analysis of Bcl-2 protein, the fluorescence intensity in the model group was significantly lower than that in the control group. We found that, after pretreatment with schisandrin, Bcl-2 fluorescence in the medium- and high-dose schisandrin groups was significantly increased compared with that in the model group. The expression level of the captopril positive control group was similar to that of the medium- and high-dose schisandrin groups. Among the different experimental groups, the fluorescence intensity of BAX protein in the model group and low-dose and high-dose schisandrin groups was stronger than that in the control group. Compared with that of the model group, the fluorescence intensity of the low-dose and high-dose schisandrin groups was similar, and the fluorescence intensity of the medium-dose schisandrin group was reduced. Therefore, schisandrin has an inhibitory effect on the JAK2/STAT3 signaling pathway but increases the fluorescence intensity of Bcl-2 and decreases the fluorescence intensity of BAX.

## 4. Discussion

We first studied the ability of schisandrin pretreatment to reduce the degree of noradrenaline-induced cardiomyocyte hypertrophy in cardiomyocytes. The results of this experiment indicated that schisandrin may inhibit the JAK2/STAT3 signaling pathway, downregulate BAX expression, upregulate Bcl-2 expression, and play a protective role in cardiomyocytes.

Cardiac hypertrophy is an independent risk factor, and it easily progresses to heart failure, which is one of the main causes of harm to human life [21]. The hypertrophic changes in cardiomyocytes are mainly characterized by an increase in cell volume and an increase in the amount of protein synthesis. Many experimental studies have shown that the JAK/STAT signaling pathway is an important signal transduction pathway often involved in the process of cardiac hypertrophy, cardiac apoptosis, and cardiac pathology [22–24]. JAK2 is of great significance in the signal transduction associated with cardiac growth and dysfunction. It can help reduce hypertrophy by inhibiting the activity of JAK2 [25]. Similarly, the activation of STAT3 causes cardiomyocyte hypertrophy and plays a role in the regulation of hypertrophic growth. The phosphorylation of STAT3 is dependent on JAK2 [26, 27]. These findings are consistent with those of our study. In NE-induced cardiac hypertrophy, the protein expression of JAK2 and STAT3 is significantly upregulated, further promoting the development of cardiomyocyte hypertrophy. When cardiomyocytes were pretreated with schisandrin and then treated with NE, the expression of JAK2 and STAT3 protein was downregulated and the surface area, protein/DNA ratio, cell viability, and apoptosis rate of cardiomyocytes were detected. The use of NE alone induced a decrease in cardiomyocyte viability. Therefore, we speculate that schisandrin protects cardiomyocytes and inhibits apoptosis by inhibiting the JAK2/STAT3 signaling pathway.

Compared with those in the model group, the myocardial cells treated with schisandrin had an increased cell survival rate and a decreased apoptosis rate. We found that the effect of schisandrin was the greatest at 10 *μ*g/mL. In addition, the ratio of BAX/Bcl-2 after pretreatment with schisandrin was reduced, and the mitochondrial membrane potential was increased. Heart failure and cardiac hypertrophy are often associated with mitochondrial dysfunction and insufficient energy, and the mitochondria play an important role in the pathogenesis of heart failure [28]. When the mitochondrial membrane is destroyed, the permeability changes, the membrane potential decreases, and the production of reactive oxygen species (ROS) increases. Consequently, a large amount of cytochrome c flows into the cytoplasm and causes apoptosis-inducing damage [29]. Studies have reported that, in rat cortical cells, schisandrin can inhibit ROS production and excessive cytochrome c release, stabilize the mitochondrial membrane potential, and downregulate caspase-3 protein expression, reducing the apoptosis rate [30]. We speculate that schisandrin may have a similar effect on the mitochondria of cardiomyocytes. The results of this experiment showed that, in NE-induced hypertrophic cells, schisandrin pretreatment induced the upregulation of Bcl-2 expression, downregulated Bax expression, decreased mitochondrial membrane potential, and decreased apoptosis. Both Bcl-2 and BAX are members of the Bcl-2 family of proteins; Bcl-2 is a protein that inhibits apoptosis, and Bax is a protein that promotes apoptosis, but they are all closely associated with the mitochondrial-mediated apoptosis pathway. Bcl-2 protein protects the mitochondrial membrane and inhibits apoptosis by inhibiting the release of cytochrome C into the cytosol [31]. The Bax protein is located in the cytoplasm. After cell damage, the BAX protein binds to Bcl-2 to form a heterodimer, which reduces the activity of Bcl-2, damages the mitochondrial membrane, and causes apoptosis [32]. Therefore, we speculate that, in this experiment, schisandrin upregulates the BAX/Bcl-2 ratio, which is related to the protection of the mitochondria. Schisandrin protects the normal function of the mitochondria, provides energy for cell functioning, inhibits cardiomyocyte apoptosis, and protects against heart failure due to hypertrophy.

In conclusion, schisandrin may reduce the ratio of BAX/Bcl-2 and inhibit the apoptosis of cardiomyocytes by inhibiting the JAK2/STAT3 signaling pathway. This research can further inform studies of the changes in other characteristics modulated by schisandrin in NE-induced cardiomyocyte hypertrophy and provide a reference for the development of new drugs for the prevention and treatment of cardiovascular diseases. However, our research is limited to the cellular level, and subsequent in vivo animal experiments are needed for validation.

## Data Availability

The data used to support the findings of this study are available from the corresponding author upon request.
